# Association of Genes Related to Oxidative Stress with the Extent of Coronary Atherosclerosis

**DOI:** 10.3390/life10090210

**Published:** 2020-09-18

**Authors:** Milena Racis, Anna Stanisławska-Sachadyn, Wojciech Sobiczewski, Marcin Wirtwein, Michał Krzemiński, Natalia Krawczyńska, Janusz Limon, Andrzej Rynkiewicz, Marcin Gruchała

**Affiliations:** 1 First Department of Cardiology, Medical University of Gdańsk, ul. Dębinki 7, 80-211 Gdańsk, Poland; wsob@gumed.edu.pl (W.S.); mgruch@gumed.edu.pl (M.G.); 2 Department of Biology and Genetics, Medical University of Gdańsk, ul. Dębinki 1, 80-211 Gdańsk, Poland; anna.stanislawska@pg.edu.pl (A.S.-S.); nataliakrawczynska@gumed.edu.pl (N.K.); jlimon@gumed.edu.pl (J.L.); 3 Department of Molecular Biotechnology and Microbiology, Gdańsk University of Technology, ul. Narutowicza 11/12, 80-233 Gdańsk, Poland; 4 Department of Pharmacology, Medical University of Gdańsk, ul. Dębinki 7, 80-211 Gdańsk, Poland; marcin.wirtwein@gumed.edu.pl; 5 Department of Probability and Biomathematics, Gdańsk University of Technology, ul. Narutowicza 11/12, 80-233 Gdańsk, Poland; mickrzem@pg.edu.pl; 6 Department of Cardiology and Cardiosurgery, University of Warmia and Mazury in Olsztyn, Al. Warszawska 30, 10-082 Olsztyn, Poland; andrzej.rynkiewicz@uwm.edu.pl

**Keywords:** oxidative stress, polymorphism, SNP, atherosclerosis, CAD, cardiovascular disease

## Abstract

Oxidative stress is believed to play a critical role in atherosclerosis initiation and progression. In line with this, in a group of 1099 subjects, we determined eight single nucleotide polymorphisms (SNPs) related to oxidative stress (*PON1* c.575A>G, *MPO* c.−463G>A, *SOD2* c.47T>C, *GCLM* c.−590C>T, *NOS3* c.894G>T, *NOS3* c.−786T>C, *CYBA* c.214C>T, and *CYBA* c.−932A>G) and assessed the extent of atherosclerosis in coronary arteries based on Gensini score. An increased risk of having a Gensini score in the higher half of the distribution was observed for the *PON1* c.575G allele (odds ratio (OR) = 1.27, 95% confidence interval (CI): 1.004–1.617, *p* = 0.046). Next, the genetic risk score (GRS) for the additive effect of the total number of pro-oxidative alleles was assessed. We noted an increase in the risk of having a Gensini score above the median with the maximum number of risk alleles (OR = 2.47, 95% CI: 1.19–5.23, *p* = 0.014). A univariate Spearman’s test revealed significant correlation between the total number of pro-oxidant alleles (GRS) and the Gensini score (ρ = 0.068, *p* = 0.03). In conclusion, the *PON1* c.575A>G variant and the high number of risk alleles (GRS) were independent risk factors for a high Gensini score. We suggest, however, that GRS might occur as a more valuable component in adding a predictive value to the genetic background of atherosclerosis.

## 1. Introduction

Atherosclerosis and its clinical manifestations still remain the main cause of death in industrialised countries [1]. The atherosclerotic process, which has traditionally been defined as a disorder of pathological lipid deposition, is now viewed as a chronic process initiated by endothelial injury that occurs in blood vessels, where smooth muscle cells proliferation and monocyte and macrophages infiltration support inflammation and local oxidation of circulating lipoproteins [2]. These processes contribute to atherosclerotic plaque formation mainly through enhancing oxidative stress within the vessel walls, which leads to further endothelial dysfunction and inflammatory response. As LDL (low-density lipoprotein) in its native form may not be atherogenic, LDL oxidation through circulating ROS (reactive oxygen species) is regarded as contributing to atherosclerotic plaque development [3,4,5]. Thus, the researchers assert that the role of oxidative stress in atherosclerosis initiation and progression is indisputable, which can make it a potential therapeutic target [6,7,8,9].

Oxidative stress can be defined as an imbalance in favour of increased levels of pro-oxidants or impaired antioxidant defence systems [10]. One of the factors that inevitably contributes to the redox state within the vasculature are pro-oxidant and antioxidant enzymes and their genetic variability. Single nucleotide polymorphisms (SNPs) within the gene sequences can inevitably exert an impact on oxidation processes, leading to atherosclerosis initiation and development. The characteristics of the enzymes investigated in the framework of this project are elucidated below, and the eight SNPs and risk alleles frequencies are listed in Table 1.

Paraoxonase 1 (PON1), which was initially described as an enzyme providing protection from pesticide poisoning, has been confirmed as playing a role in a wide variety of human illnesses, including cardiovascular disease [11,12,13]. As PON1 prevents LDL oxidation and degrades oxidized lipids, decreased PON1 expression has been reported in atherosclerotic arteries [14]. Regarding the *PON1* c.575A>G (Gln192Arg) variant (rs662), the G allele has been associated with either higher paraoxonase activity [15] or lower plasma arylesterase activity [14]. Interestingly, PON1 Gln192Arg variants vary in their rate of metabolism of different substrates [16]. Among the *PON1* c.575GG carriers, the presence of hypertriglyceridemia reduced, to a greater degree, blood vessel reactivity [17]. Further, the *PON1* c.575G allele was referred to as a risk allele in some clinical studies concerning cardiovascular diseases [14,18,19].

Myeloperoxidase (MPO) is an enzyme expressed in neutrophils and monocytes, where it generates a potent oxidant, hypochlorous acid (HOCI), which is an important element of an immune defence mechanism [20]. However, its pro-oxidative function means it is also involved in LDL oxidation and ROS production within atherosclerotic plaques [21,22]. The G allele of the *MPO* c.−463G>A polymorphism (rs 2333227), located in the SP-1 transcription factor binding site, has been linked to increased enzyme expression [23], although some studies did not confirm that finding [24].

Manganese superoxide dismutase (MnSOD), encoded by the *SOD2* gene, is an antioxidant enzyme catalysing the dismutation of reactive superoxide radicals to hydrogen peroxide [25]. The *SOD2* c.47T>C polymorphism (rs4880) within exon 2 results in an amino acid substitution Val16Ala, which causes the less efficient transport of MnSOD into the mitochondrion, which leads to impaired intramitochondrial ROS neutralization [26,27].

Glutathione is one of the main intracellular antioxidants, as it regulates the redox state within the cell, protecting it from oxidative injury [28,29]. Glutamate-cysteine ligase (GCL), which consists of a catalytic subunit (GCLC) and a modifier subunit (GCLM), is a rate-limiting enzyme synthesizing glutathione [30]. The T allele of the c.−590C>T polymorphism (rs41303970) within the *GCLM* promoter is associated with lower oxidant-induced *GCLM* gene expression and lower plasma glutathione levels [31].

Synthesized by endothelial nitric oxide synthase (eNOS), nitric oxide has been reported to inhibit lipid peroxidation in the vasculature and also cause smooth muscle relaxation, thus resulting in vasodilation and increasing blood flow [32,33]. The 894T allele of *eNOS c.*894G>T polymorphic site (rs1799983) leads to an amino acid Glu298Asp substitution and results in higher susceptibility to proteolysis enzymes [34,35]. The *eNOS c.*−786T>C polymorphism (rs2070744) has been reported to reduce promoter activity by about 50% [36]. In consequence, both of these allelic changes lead to lower eNOS enzymatic function, which results in diminished nitric oxide production [37,38].

The enzyme primarily responsible for the oxidative burst in neutrophils is NADPH oxidase, a multi-unit cytochrome complex that generates ROS extracellularly, which leads to the elimination of invading microorganisms. However, it also mediates diverse functions in various organisms intracellularly through redox signalling [39]. There are several isoforms of NADPH oxidase (NOX1-NOX5), while NOX2 is found in the vasculature. NOX are enzymatic complexes that differ in the types of subunits of which they consist. However, they all contain the regulatory subunit-p22Phox, encoded by the *CYBA* gene, crucial for the activity of the whole enzymatic complex [40]. The *CYBA* c.214 T allele (rs4673) has been associated with higher NADPH oxidase enzymatic activity; as a result, it is defined as a risk factor for atherosclerosis and brain stroke [41,42]. The G allele within the *CYBA* c.−932A>G polymorphic site (rs9932581), located in a potential binding site for transcription factors, has been reported to exhibit increased expression of p22Phox and thus lead to increased ROS production [43].

Therefore, the aim of our study was to investigate the association between the eight SNPs located in genes related to oxidative stress, *PON1* c.575A>G, *MPO* c.−463G>A, *SOD2* c.47T>C, *GCLM* c.−590C>T, *NOS3* c.894G>T, *NOS3* c.−786T>C, *CYBA* c.214C>T, and *CYBA* c.−932A>G, and the extent of atherosclerosis, as quantified by Gensini score, which is a scientific tool for measuring the amount of atherosclerosis in coronary arteries. Thus, individual analysis of each selected SNP was performed, followed by analyses of genetic risk score (GRS), considering the total number of pro-oxidative alleles within polymorphic sites of the eight SNPs investigated.

## 2. Results

### 2.1. Individual Oxidative Stress-Related Polymorphisms and Coronary Atherosclerosis

The general characteristics of the studied population (n = 1099) and two subgroups based on a median value of Gensini score (with Gensini score either below or above the median) are presented in Table 1, which also contains risk alleles frequencies.

There were no significant differences between these subgroups except for the percentage of men and percentage of smokers, which were higher in the group with Gensini ≥40, and the HDL level, which was lower in this group. All the SNPs were in Hardy–Weinberg equilibrium.

A statistically significant increase in the risk of more severe atherosclerosis (that is, of having the Gensini score in the higher half of the distribution) was observed for the *PON1* c.575G allele carriers (odds ratio (OR) = 1.27, 95% confidence interval (CI): 1.004–1.617, *p* = 0.046). A risk of having a higher Gensini score increased when the number of risk alleles increased: AA (ref.) vs. AG (OR = 1.23, 95% CI: 0.96–1.58) vs. GG (OR = 1.55, 95% CI: 0.95–2.55, *p* for trend = 0.028) (Table 2).

Similarly, the univariate analysis and Spearman’s rank correlation showed that the Gensini score was significantly correlated with the increasing number of risk alleles in the *PON1* (ρ = 0.069, *p* = 0.023) (Table 3).

Furthermore, the median of the Gensini parameter was significantly higher in the group of patients with the GG genotype than among those with an A allele (50, Q_1_–Q_3_ = 25–80 vs. 40, Q_1_–Q_3_ = 16–68, *p* = 0.023); this significance remained after correction for multiple comparisons (dominant genetic model) (Table S1).

A statistically significant increase in the risk of being in the higher half of the Gensini score distribution was observed among the *MPO* c.−463AA genotype carriers (OR = 2.69, 95% CI: 1.12–6.45, *p* = 0.021); however, the *p* value for the trend as the number of risk alleles increased was non-significant (Table 2). Moreover, although Gensini medians showed significant differences in a dominant model analysis comparing the AA genotype carriers and the G allele carriers (40, Q_1_–Q_3_ = 16–69 vs. 51, Q_1_–Q_3_ = 38–81, *p* = 0.048), they did not reach statistical significance after correction for multiple comparisons (Table S1). In analyses regarding the *SOD2* c.47T>C polymorphism, no statistically significant increase in the risk of having a higher Gensini score as the number of risk alleles increased was observed. Although, regarding Gensini score, significant differences in medians were observed when comparing the *SOD2* c.47TT genotype group vs. the C allele group (44.0, Q_1_–Q_3_ = 20–74 vs. 38.5, Q_1_–Q_3_ = 15–68, *p* = 0.044), the differences did not reach statistical significance following a Bonferroni correction for multiple comparisons. Likewise, we found no statistically significant associations regarding Gensini score and the *GCLM* c.−590C/T, *NOS3* c.894G/T, *NOS3* c.−786T/C, *CYBA* c.214C>T, and *CYBA* c.−932A>G genotypes (Table 2, Table S1).

In the univariate Spearman’s analysis, Gensini score was positively correlated with male sex (ρ = 0.151, *p* < 0.0001), triglycerides (ρ = 0.074, *p* = 0.021), and history of smoking (ρ = 0.093, *p* = 0.003) and inversely correlated with HDL cholesterol (ρ = −0.116, *p* = 0.0003), as shown in Table 3. To test whether the *PON1* c.575A>G polymorphism is independently associated with Gensini score, a stepwise multivariate linear analysis was performed. The results indicated that the *PON1* c.575 GG genotype (β = 0.402, *p* = 0.015), age (β = 0.031, *p* = 0.008), gender (β = 1.088, *p* < 0.0001), LDL cholesterol level (β = −0.006, *p* = 0.015), HDL cholesterol level (β = −0.022, *p* = 0.005), and triglycerides level (β = 0.563, *p* = 0.022) were independent risk factors for a high Gensini score (Table 3, *PON1* Model).

Our results suggest that the *MPO* c.−463AA and *PON1* c.575GG genotypes increase the risk of more severe atherosclerosis. There were also differences in medians in groups defined by the *PON1* c.575A>G, *MPO* c.−463G>A, and *SOD2* c.47T>C genotypes in dominant and additive model analyses, but following the Bonferroni corrections, only the *PON1* c.575A>G remained significant and was indicated to be independently associated with the extent of coronary atherosclerosis.

### 2.2. The Association of Eight Oxidative Stress-Related SNPs and Coronary Atherosclerosis 

**Figure 1:**
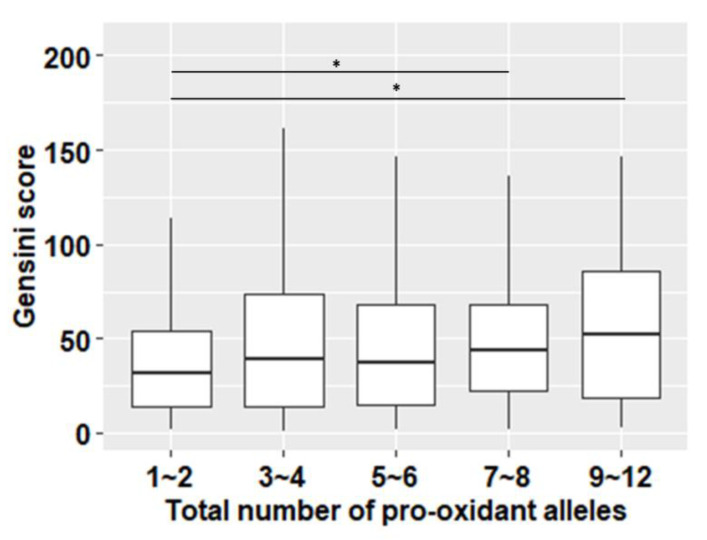
Values of Gensini score (Q_1_–Q_3_) for subjects with 1–12 pro-oxidant alleles. Significant differences in Gensini scores between subjects with 1~2 risk alleles and those with 7~8 and 9~12 risk alleles were observed (*p* < 0.05, Kruskal–Wallis test, followed by Dunn’s Multiple Comparison Test).

To evaluate the additive effects of the eight studied SNPs on the Gensini score, the subjects were divided into groups according to the number of pro-oxidant alleles they carried within each SNP (0: no pro-oxidant allele; 1: one pro-oxidant allele, 2: two pro-oxidant alleles) multiplied by 8 (the number of SNPs). Although the theoretical number of groups ranged from 0 (none risk alleles) to 16 (exclusively risk alleles present), we selected 12 groups of patients defined by the number of pro-oxidative alleles: beginning from the group with one risk allele to the group with 12 risk alleles: 1 (n = 16), 2 (n = 50), 3 (n = 114), 4 (n = 180), 5 (n = 188), 6 (n = 211), 7 (n = 134), 8 (n = 79), 9 (n = 40), 10 (n = 9), 11 (n = 5), 12 (n = 1). There were no patients with 0, 13, 14, 15, or 16 risk alleles. Only the subjects with complete genetic data were considered in these analyses. Those 12 groups were classified into five GRS groups by two: 1~2, 3~4, 5~6, 7~8 and by four: 9~12, since the number of subjects within the last groups was exceptionally low. Gensini medians (Q_1_–Q_3_) and patients’ numbers within GRS groups were as follows: 31.5 (Q_1_–Q_3_ = 13.5–53.8), n = 66, 39.5 (Q_1_–Q_3_ = 14–73.8), n = 294, 37 (Q_1_–Q_3_ = 15–68) n = 399, 44 (Q_1_–Q3 = 22–68), n = 213, and 52 (Q_1_–Q_3_ = 19–86), n = 55, respectively. There were significant differences in Gensini medians regarding the group with the lowest number of pro-oxidant alleles and the two groups with the highest number of pro-oxidant alleles: 1~2 vs. 7~8 (*p* = 0.018) and 1~2 vs. 9~12 (*p* = 0.017) (Figure 1, Table S1). We did not notice statistical differences in Gensini medians while comparing other GRS groups.

We also observed a statistically significant increase in the risk of having a Gensini score above the median regarding GRS group with 7~8 risk alleles (OR = 1.98, 95% CI: 1.13–3.51, *p* = 0.016) and GRS group with 9~12 risk alleles (OR = 2.47, 95% CI: 1.19–5.23, *p* = 0.014), when the 1~2 GRS group was treated as a reference (Table 2). A univariate analysis (Spearman’s rank correlation coefficient test) revealed that there was a significant correlation between the total number of pro-oxidant alleles (GRS) and Gensini score (ρ = 0.068, *p* = 0.03) (Table 3). To establish whether the number of pro-oxidant alleles is a determinant to high Gensini value independently of other risk factors, a forward and backward stepwise multivariate regression linear analysis was executed using all the clinical variables available in our study. We constructed a model in which the following variables were found to have a descriptive value: age, male sex, LDL cholesterol, high-density lipoprotein (HDL) cholesterol, and triglycerides level. Our analyses show that the number of risk alleles (GRS) (β = 0.133, *p* = 0.014), age (β = 0.031, *p* = 0.01), male sex (β = 1.054, *p* < 0.0001), LDL cholesterol (β = −0.005, *p* = 0.03), HDL cholesterol (β = −0.02, *p* = 0.014), and triglycerides (β = 0.739, *p* = 0.004) were independent risk factors for having a high Gensini score (Table 3, GRS Model).

The results of our study indicate that GRS, defined as the accumulation of eight studied pro-oxidative alleles, is significantly associated with the extent of coronary atherosclerosis quantified by Gensini score, independent of other conventional risk factors.

## 3. Discussion

Oxidative stress is believed to play a critical role in the initiation and progression of atherosclerosis. Since the oxidative stress level within the vasculature can be partly determined by genetic background, we presumed that enzymes related to oxidative stress would prove to be a fruitful target of investigation due to polymorphic sites that influence their expression and function. Functional analyses showed that levels of serum 8-hydroxy-2′-deoxyguanosine (8-OHdG), an indicator of oxidative stress, increase with increasing numbers of pro-oxidative alleles [31].

Therefore, in this study, we determined eight relatively common genetic variants related to oxidative stress and examined their association with the coronary atherosclerosis quantified by the Gensini score. We determined pro-oxidative alleles (risk alleles for atherosclerosis) for each SNP on the basis of their functional effect [44,45,46] and based on clinical research regarding populations with cardiovascular disease [42,47,48].

Initially, we performed analyses wherein the association between each SNP and the Gensini score was examined individually, which showed only the *PON1* c.575A>G polymorphism being independently associated with the extent of atherosclerosis. Paraoxonase 1, encoded by the *PON1* gene, is found in many tissues and in circulation, and as a protein is bound to high-density lipoproteins (HDL). It is also capable of degrading oxidized lipids in LDL lipoproteins [13,49,50,51,52]. Interestingly, the *PON1* c.575A allele is associated with lower paraoxonase 1 activity and can be defined as a risk allele for developing a disease state, as functional and clinical studies report [15,18,47,53]. However, some other researchers do not confirm this hypothesis [54,55,56], or even claim the opposite [57,58,59,60]. In the context of these findings, the association between individual polymorphisms and cardiovascular disease remains controversial, and it is now accepted that the effect of an individual polymorphism may not be strong enough to explain the disease occurrence [61,62,63,64].

Considering the above, we conclude that even though the results of our study indicate that one of the studied SNPs has a potential clinical impact on some aspects of cardiovascular disease, it is noteworthy to determine the additive effect of all eight genetic variants, as such an accumulation could substantially influence oxidative stress level, which in turn may affect atherosclerosis development. Therefore, based on the working hypothesis that accumulation of several genetic polymorphisms can substantially have an impact on the coronary artery disease, we determined the combined effect of the eight selected SNPs in the context of atherosclerosis. A statistically significant increase in the risk of more severe atherosclerosis was observed for the individuals with the highest amount of pro-oxidative alleles, and the analyses showed GRS as an independent risk factor for a high Gensini value (Table 3, GRS Model). These results indicate that, although some genes seem to exert a more significant impact on the disease, it is legitimate to examine several SNPs combined when we plan to estimate the potential risk of an individual getting a disease, especially considering the multigenetic disorders wherein cardiovascular disease is one of the representatives. As a result, this may indicate better routes in a genetic approach to the clinical management of cardiovascular disease.

Our study, however, had several limitations. First, since the number of subjects with the highest number of pro-oxidative alleles was exceptionally low, we had to combine the last four groups to analyse them as one. To overcome this effect, larger trials would be necessary. Secondly, in some cases, it is hard to translate the functional effect of selected SNPs on disease origination and progression. Although for our study we chose only the SNPs with a known functional effect, the true molecular mechanisms regarding how those genes’ accumulation influences the oxidative stress level and, in consequence, the extent of coronary atherosclerosis, should be more profoundly evaluated in further functional studies. It is also notable that in our analyses we did not take into consideration the number of significant atherosclerotic narrowings in coronary arteries, which is important from a clinical perspective in order to qualify a patient for further invasive treatment. However, the aim of our study was to examine the impact of genetic factors on the extent of atherosclerosis as a whole, despite the clinical significance of coronary narrowings. The Gensini score, in comparison with other clinical scales, quantifies in detail even mild coronary atherosclerotic lesions, as it takes into account the number of narrowings’ and the degree of the coronary segment importance. Finally, since the extent of atherosclerosis in coronary arteries does not always correlate with major adverse cardiovascular events (MACE), further prospective analyses would be highly valuable to assess the additive effect of these SNPs on death, acute coronary syndromes, or cardiovascular rehospitalisation.

## 4. Materials and Methods

### 4.1. Study Population

**Figure 2:**
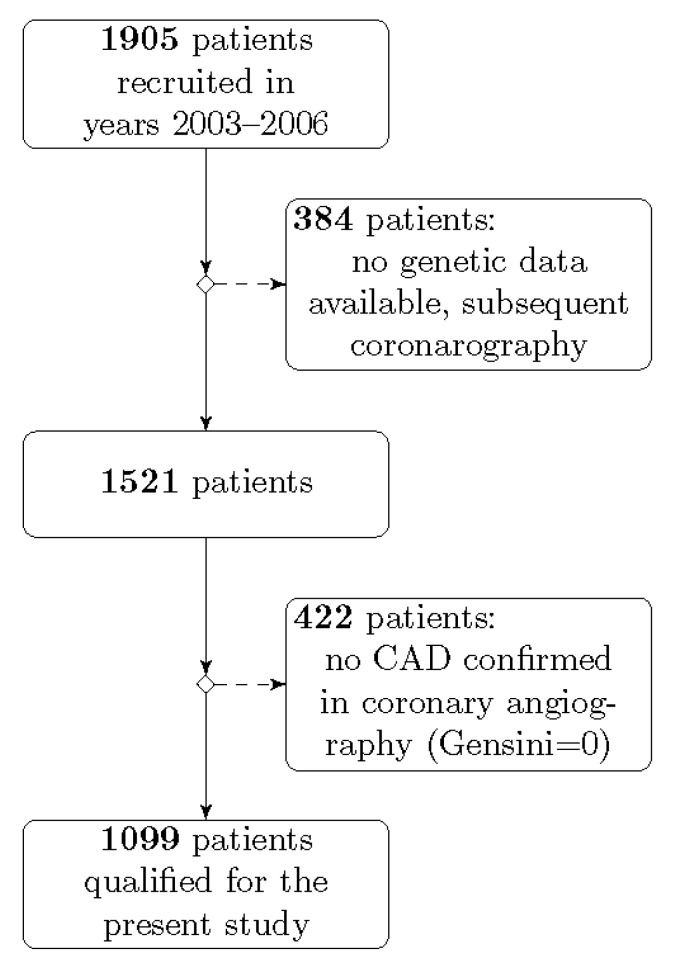
The inclusion and exclusion criteria of the patients recruited into the study.

The study population was constructed in the framework of the project conducted in the First Department of Cardiology, Medical University of Gdansk between 2003 and 2006. Patients enrolled in this project had clinical symptoms of coronary artery disease (CAD) or positive non-invasive tests (exercise stress test, computed tomography angiogram, or scintigraphy of the heart), which qualified them for planned coronary angiography. The database was previously used in other genetic and non-genetic studies [42,65,66,67]. After considering the inclusion and exclusion criteria, 1099 individuals out of 1905 were recruited for the present study (Figure 2).

The inclusion criteria were as follows: age of at least 18 years, presence of atherosclerotic lesions confirmed in coronary angiography, and molecular data containing at least one of the investigated polymorphic sites. Exclusion criteria were the following: lack of the patient’s consent, absence of atherosclerotic lesions in coronary arteries, no genetic data available, and no first coronarography data available (patients undergoing subsequent coronarography).

On admission day, the clinical interview was conducted. Hypertension was diagnosed either on the basis of blood pressure measurements during hospitalization or based on whether the patient was on hypotensive therapy. Diabetes was diagnosed based on fasting glucose levels, oral glucose tolerance test, or use of hypoglycaemic therapy. Smoking status was self-reported. Fasting blood samples were collected for biochemical tests. Additionally, whole blood samples were collected to isolate DNA. The protocol of this study was approved by the Ethics Committee of the Medical University of Gdansk (Ref No. NKBBN/486/2012 issued on 12 December 2012) and conforms to the ethical guidelines of the 1964 Declaration of Helsinki.

### 4.2. Coronary Atherosclerosis Evaluation

To assess the extent of atherosclerosis in coronary arteries and determine the need for invasive treatment, each subject had coronary angiography performed, a procedure that, due to X-ray contrast injection, enables visualization of the coronary artery tree. The number and extent of the atherosclerotic lesions were evaluated by two independent invasive cardiologists. Gensini score was applicated as a tool for measuring the extent of atherosclerosis using the medical calculator from The Medical Algorithms Project. Every atherosclerotic narrowing from 25% to 100% received punctuation, which was subsequently multiplied by a Gensini ratio depending on segment importance and its location on a coronary artery tree (significance of jeopardized myocardium) [68,69]. Only the individuals with coronary atherosclerosis presence (Gensini score ≥1), independently of clinical significance, were enrolled, whereas subjects with no atherosclerotic changes in coronary arteries were excluded.

### 4.3. Genetic Analyses

In this study, eight SNPs located in genes associated with oxidative stress were selected: *PON1* c.575A>G, *MPO* c.−463G>A, *SOD2* c.47T>C, *GCLM* c.−590C>T, *NOS3* c.894G>T, *NOS3* c.−786T>C, *CYBA* c.214C>T, and *CYBA* c.−932A>G.

DNA was isolated from venous blood lymphocytes using a fenol-chloroform method and A&A Biotechnology Kit (Genomic Maxi AX, 995-10) according to the manufacturer’s protocol. DNA quality was examined using a NanoDrop spectrophotometer (Thermo Scientific, Waltham, MA, USA). Genotyping within seven analysed loci were determined using a 5′nuclease Real-Time PCR assay on LightCycler 480 (Roche, Basel, Switzerland). Positive controls representing all genotype classes and a negative control were included in each plate. Dual fluorescence was detected after each completed cycle. Each sample was genotyped in duplicate, and the validation of the results was performed via bidirectional sequencing of all investigated variants. PCR products were sequenced using a BigDye Terminator v3.1 Cycle Sequencing Kit and 3100 Series Genetic Analyzer (Thermo Fisher Scientific). Electropherograms were analysed with a Sequencer v.10 DNA Software (Gene Codes, Ann Arbor, MI, USA). Primers, probe sequences, concentrations of reagents, and genotyping conditions are listed in Table S2.

### 4.4. Statistical Analysis

Distributions of continuous variables were expressed as means ± SD (standard deviation) or medians (Q_1_–Q_3_). Differences in Gensini levels between three genotype classes were assessed using a Kruskal–Wallis test, while differences between two groups in dominant or recessive models were evaluated using a Wilcoxon test. In those comparisons, Bonferroni’s correction was applied to yield the corrected level of significance, namely 0.025 (=0.05/2). A Kruskal–Wallis test, followed by Dunn’s Multiple Comparison Test, was applied to compare Gensini values between the GRS groups. To determine the risk scores (OR; 95% CI) using logistic regression, we divided the population into two groups according to the Gensini median value (median = 40). We then assessed the prevalence of individual genotypes and GRS in groups defined as Gensini score <40 and Gensini score ≥40. Univariate analysis and Spearman’s rank correlation were used as a measure of the strength of association, and the direction of the relationship between the Gensini score and conventional and genetic variables. Multiple linear regression models, with the Gensini score as the response variable, were created. In the PON1 Model, the analysis included the PON1 c.575A>G genotypes as a genetic variable, and in the GRS Model the combined effect of all the eight SNPs constituted a genetic variable. The Gensini score was transformed using a Box–Cox transformation in those models. To correct for non-linearity and/or unequal variances, the predictor variable (triglycerides level) was log-transformed. A bidirectional elimination approach to stepwise multivariate regression was employed to evaluate the relationship between the transformed Gensini score and the following variables: age, gender, BMI, presence of hypertension and diabetes, lipid profile, and smoking status. To construct the multiple regression model, we chose only those variables that explained the largest Gensini score variability; in our study, apart from genetic factors, those conventional variables were age, gender, LDL cholesterol, HDL cholesterol, and triglycerides level. Clinical characteristics of the patients were presented as means ±SD and medians for continuous variables (i.e., BMI, triglycerides level) or as percentages for categorical variables (i.e., smoking history, hypertension, diabetes). Smoking status was self-reported.

Deviations from the Hardy–Weinberg equilibrium for the genotypes were assessed using a chi-square test. The chi-square tests were used to compare frequencies of categorical variables between groups, and the Wilcoxon test was used to compare levels of continuous variables between groups. The level of statistical significance was set at *p* < 0.05 (if not indicated otherwise). A statistical analysis was performed using R Statistical Software Package (Foundation for Statistical Computing, Vienna, Austria).

## 5. Conclusions

The results of our study indicate that both *PON1* c.575A>G and GRS, defined as the accumulation of pro-oxidative alleles, are suggested to exert an impact on the extent of atherosclerosis in coronary arteries, independent of other conventional risk factors. However, in the context of our own findings and based on previous research regarding SNPs in CAD, we suggest that GRS might occur as a more valuable component in adding a predictive value to the genetic background of atherosclerosis.

## Figures and Tables

**Table 1:** Patients characteristics with Gensini score below (<40) and above median (≥40) and risk alleles frequencies.

Parameters		Total	Gensini <40	Gensini ≥40	*p* Value
		n = 1099	n = 540 (49.1%)	n = 559 (50.9%)	
Age (years): mean ± SD		64.2 ± 9.4	63.9 ± 9.5	64.5 ± 9.4	ns ^a^
median (min, max), n		65 (36, 87), 1094	64.5 (36, 87), 538	65.5 (36, 84), 556	
Male: n (%)		726 (66.1%)	327 (60.6%)	399 (71.5%)	0.0001 ^b^
BMI (kg/m^2^): mean ± SD		28.0 ± 4.1	27.9 ± 4.1	28.1 ± 4.0	ns ^a^
median (min, max), n		27.7 (14.9, 42.8), 1047	27.7 (14.9, 42.8), 516	27.7 (16.6, 40.9), 531	
Hypertension: n (%)		856 (81.1%)	419 (80.7%)	438 (81.4%)	ns ^b^
Diabetes: n (%)		265 (25.0%)	132 (25.3%)	133 (24.7%)	ns ^b^
Total cholesterol (mg/dL): mean ± SD		207.1 ± 51.6	206.8 ± 50.1	207.3 ± 53.0	ns ^a^
median (min, max), n		198 (92, 552), 983	198 (98, 417), 486	198 (92, 552), 497	
LDL cholesterol (mg/dL): mean ± SD		123.3 ± 43.8	122.4 ± 43.4	124.2 ± 44.3	ns ^a^
median (min, max), n		116 (7, 452), 905	115 (7, 317), 447	117 (37, 452), 458	
HDL cholesterol (mg/dL): mean ± SD		54.8 ± 14.5	56.6 ± 16.1	52.9 ± 12.4	0.001 ^a^
median (min, max), n		52 (26, 175), 956	53 (26, 175), 480	51 (26, 141), 489	
Triglycerides (mg/dL): mean ± SD		147.2 ±94.8	144.8 ± 98.4	149.5 ± 91.1	ns ^a^
median (min, max), n		124 (36, 897), 975	122.5 (42, 897), 482	128 (36, 878), 493	
History of smoking: n (%)		678 (67.0%)	316 (63.2%)	362 (70.7%)	0.011 ^b^
CAD in family: n (%)		546 (54%)	267 (53.9%)	279 (54.0%)	ns ^b^
Coronary narrowings ≥50% n (%)		1070 (97.4%)	511 (94.6%)	559 (100%)	<0.0001 ^b^
Coronary narrowings ≥70% n (%)		990 (90.1%)	431 (79.8%)	559 (100%)	<0.0001 ^b^
SNP	Risk allele	Risk allele frequency			
*PON1* c.575 A>G	G	0.270	0.250	0.291	0.035 ^b^
*MPO* c.−463 A>G	A	0.164	0.157	0.170	ns ^b^
*SOD2* c.47C>T	T	0.476	0.462	0.490	ns ^b^
*GCLM* c.588 C>T	T	0.165	0.166	0.165	ns ^b^
*eNOS* c.894 G>T	T	0.277	0.269	0.284	ns ^b^
*eNOS* c.−786 C>T	C	0.364	0.352	0.377	ns ^b^
*CYBA* c.214 T>C	T	0.346	0.342	0.351	ns ^b^
*CYBA* c.−930 G>A	G	0.596	0.590	0.602	ns ^b^

ns: non statistically significant; n: number of subjects included into analysis; SD: standard deviation; BMI: Body mass index; LDL: low-density lipoprotein; HDL: high-density lipoprotein; CAD: coronary artery disease; SNP: single nucleotide polymorphism; ^a^—Wilcoxon rank-sum test, ^b^—Pearson’s chi-squared test.

**Table 2:** Odds ratios of Gensini scores ≥40 occurrence in relation to selected genotypes and genetic risk score (GRS) groups.

SNPs/GRS	Genotype/GRS Group	Gensini Score <40 (% of Genotype Carriers)	Gensini Score ≥40 (% of Genotype Carriers)	OR (95% CI), *p*
rs662 *PON1* c.575A>G	AA	299 (55.7)	275 (49.6)	1 (Ref)
	AG	208 (38.7)	236 (42.6)	1.23 (0.96–1.58), *p* = 0.097
	GG	30 (5.6)	43 (7.8)	1.55 (0.95–2.55), *p* = 0.076
				*p* for trend = **0.028**
	A allele (ref) vs. GG			1.42 (0.88–2.30), *p* = 0.151
	AA (ref) vs. G allele			1.27 (1.004–1.617), *p* = **0.046**
rs2333227 *MPO* c.−463A>G	GG	375 (69.8)	385 (69.5)	1 (Ref)
	GA	155 (28.9)	150 (27.1)	0.94 (0.72–1.22), *p* = 0.663
	AA	7 (1.3)	19 (3.4)	2.64 (1.10–6.36), *p* = **0.025**
				*p* for trend = 0.432
	A allel vs. GG (ref)			1.02 (0.78–1.31), *p* = 0.903
	AA vs. G allel (ref)			2.69 (1.12–6.45), *p* = **0.021**
rs4880 *SOD2* c.47C>T	CC	148 (27.6)	147 (26.5)	1 (Ref)
	CT	282 (52.5)	271 (48.9)	0.97 (0.73–1.28), *p* = 0.828
	TT	107 (19.9)	136 (24.6)	1.28 (0.91–1.79), *p* = 0.156
				*p* for trend = 0.183
	T allele vs. CC (ref)			1.05 (0.81–1.37), *p* = 0.703
	TT vs. C allele (ref)			1.31 (0.98–1.74), *p* = 0.067
rs41303970 *GCLM* c.588C>T	CC	376 (70.0)	387 (69.9)	1 (Ref)
	CT	144 (26.8)	151 (27.2)	0.99 (0.77–1.28), *p* = 0.945
	TT	17 (3.2)	16 (2.9)	1.13 (0.76–1.66), *p* = 0.548
				*p* for trend = 0.972
	T allele vs. CC (ref)			1.00 (0.78–1.31), *p* = 0.953
	TT vs. C allele (ref)			0.91 (0.45–1.82), *p* = 0.789
rs1799983 *eNOS* c.894G>T	GG	290 (53.8)	282 (51.0)	1 (Ref)
	GT	208 (38.6)	228 (41.2)	1.13 (0.88–1.45), *p* = 0.346
	TT	41 (7.6)	43 (7.8)	1.08 (0.68–1.71), *p* = 0.746
				*p* for trend = 0.437
	GG (ref) vs. T allele			1.12 (0.88–1.42), *p* = 0.353
	G allele (ref) vs. TT			1.02 (0.66–1.60), *p* = 0.917
rs2070744 *eNOS* c.−786C>T	TT	223 (42.7)	214 (40.1)	1 (Ref)
	CT	231 (44.3)	236 (44.3)	1.06 (0.82–1.38), *p* = 0.638
	CC	68 (13.0)	83 (15.6)	1.27 (0.88–1.84), *p* = 0.204
				*p* for trend = 0.232
	CC vs. T allele (ref)			1.23 (0.87–1.74), *p* = 0.238
	C allele vs. TT (ref)			1.11 (0.87–1.42), *p* = 0.397
rs4673 *CYBA* c.214T>C	CC	230 (43.3)	234 (42.9)	1 (Ref)
	CT	239 (45.0)	241 (44.1)	0.99 (0.77–1.28), *p* = 0.945
	TT	62 (11.7)	71 (13.0)	1.13 (0.76–1.66), *p* = 0.548
				*p* for trend = 0.666
	T allele vs. CC (ref)			1.02 (0.80–1.30), *p* = 0.880
	TT vs. C allele (ref)			1.13 (0.79–1.63), *p* = 0.508
rs9932581 *CYBA* c.−930G>A	AA	84 (16.0)	93 (17.0)	1 (Ref)
	AG	262 (50.0)	248 (45.4)	0.85 (0.61–1.2), *p* = 0.369
	GG	178 (34.0)	205 (37.6)	1.04 (0.73–1.49), *p* = 0.828
				*p* for trend = 0.546
	A allele (ref) vs. GG			1.17 (0.91–1.50), *p* = 0.222
	AA (ref) vs. G allele			0.93 (0.67–1.28), *p* = 0.659
Genetic Risk Score (GRS)	1~2	40 (7.9)	26 (5.0)	1 (Ref)
	3~4	147 (29.1)	147 (28.2)	1.53 (0.89–2.67), *p* = 0.119
	5~6	205 (40.5)	194 (37.2)	1.45 (0.86–2.50), *p* = 0.164
	7~8	93 (18.4)	120 (23.1)	1.98 (1.13–3.51), *p* = **0.016**
	9~12	21 (4.1)	34 (6.5)	2.47 (1.19–5.23), *p* = **0.014**
				*p* value for trend **0.008**

T allele = TT genotype + CT genotype; C allele = CC genotype + CT genotype; A allele = AA genotype + AG genotype; G allele = GG genotype + AG genotype; OR: odds ratio; CI: confidence interval. Bold font indicates statistical significance.

**Table 3:** Association between risk factors and Gensini score.

PARAMETERS	GENSINI SCORE					
	Univariate Analysis		Multivariate Regression Analysis			
	Spearman’s Rank Correlation Rho ρ	*p* Value	*PON1* Model		GRS Model	
			β	*p* Value for F test	β	*p* Value for F test
Age (years)	**0.038**	0.212	0.031	**0.004**	0.031	**0.006**
Male sex (%)	0.151	<0.0001	0.965	**<0.0001**	0.907	**<0.0001**
BMI (kg/m^2^)	−0.008	0.787	-	**-**	-	-
Hypertension (%)	−0.019	0.535	-	**-**	-	-
Diabetes (%)	−0.020	0.523	-	**-**	-	-
Total cholesterol (mg%)	0.013	0.673	0.007	**0.001**	0.007	**0.003**
LDL cholesterol (mg%)	0.039	0.236	-	**-**	-	-
HDL cholesterol (mg%)	−0.116	0.0003	−0.036	**<0.0001**	−0.037	**<0.0001**
Triglycerides (mg%)	0.074	0.021	-	**-**	-	-
History of smoking (%)	0.093	0.003	-	**-**	-	-
*PON1* c.575A>G	0.069	0.022	0.43	**0.007**	NA	NA
GRS	0.068	0.030	NA	NA	0.128	**0.014**

ns: non-significant; NA: non-analysed; BMI: Body mass index; LDL: low-density lipoprotein cholesterol; HDL: high-density lipoprotein cholesterol; CAD: coronary artery disease; β: partial regression coefficient, GRS: genetic risk score (total number of risk alleles); *PON1* c.575A>G: AA = 0, AG = 1, GG = 2; Variables were considered for the multivariate regression model when their simple linear regression *p* value was < 0.05. While applying a bidirectional elimination approach to stepwise multivariate regression, LDL cholesterol and triglyceride level were excluded due to severe multicollinearity with the total cholesterol; BMI, hypertension, diabetes, and smoking status were excluded and found not to be confounding variables in our models; *p* value for F test is the *p*-value for ANOVA to test whether the more complex model (including given variable) is significantly better than the simpler model (excluding given variable). Bold font indicates statistical significance.

## Supplementary Materials

The following are available online at https://www.mdpi.com/2075-1729/10/9/210/s1, Table S1. The medians of the Wilcoxon rank sum test and means with standard deviation for Gensini score in dominant, recessive, and additive models of the eight studied SNPs. Table S2. Genotyping conditions, primers, and probes sequences.

## Abbreviations

SNP	single nucleotide polymorphism
GRS	genetic risk score
CAD	coronary artery disease
ROS	reactive oxygen species
BMI	body mass index
LDL	low density lipoprotein
HDL	high density lipoprotein
SD	standard deviation
MACE	major adverse cardiovascular events
