# Supplementary material for: Association of Genes Related to Oxidative Stress with the Extent of Coronary Atherosclerosis
Source: Life (Basel). 2020 Sep 18;10(9):210. doi: 10.3390/life10090210 (PMC7554836; doi:10.3390/life10090210)
Supplement: Supplementary file 1 [file life-10-00210-s001.pdf]

life

MDPI

Table S1. Median (interquartile ranges), mean (standard deviation) of Gensini score defined by genotypes, in dominant, recessive and additive models, of the eight studied SNPs. Significance was determined by the Wilcoxon rank sum test.

Single Nucleotide Polymorphisms	Genetic Models	N	Gensini Median (Q_1-Q_3)	Gensini Mean (SD)	*p* Value
PON1 c.575A>G	A allele vs GG[D]	1018 vs 73	40 (16-68) vs 50 (25-80)	47.07(37.8) vs 57.3 (40.4)	**0.023**[B]
	G allele vs AA[R]	517 vs 574	42 (19-71) vs 37 (14.25-68)	49.74 (38.59) vs 45.97 (37.49)	ns
	AA vs AG vs GG[A]	574 vs 444 vs 73	37 (14.25-68) vs 42 (16.75-68) vs 50 (25-80)	45.97 (37.49) vs 48.5 (38.20) vs 57.30 (40.36)	**0.037**[k]
MPO c.-463A>G	G allele vs AA[D]	1065 vs 26	40(16-69) vs 51 (37.75-81.25)	47.45(37.98) vs 60.62 (40)	**0.048**
	A allele vs GG[R]	331 vs 760	40 (16-66) vs 40 (16-70)	48.03 (29.46) vs 47.64 (37.47)	ns
	GG vs GA vs AA[A]	760 vs 305 vs 26	40 (16-70) vs 38 (14-66) vs 51 (37.75-81.25)	47.64 (37.47) vs 46.96 (39.29) vs 60.62 (40)	ns[k]
SOD2 c.47T>C	C allele vs TT[D]	848 vs 243	38.5 (15-68) vs 44.0 (20-74)	46.6(37.7) vs 51.8 (39.1)	**0.044**
	T allele vs CC[R]	796 vs 295	40 (16-71) vs 38 (16-66)	48.61 (38.38) vs 45.46 (37.11)	ns
	CC vs CT vs TT[A]	295 vs 553 vs 243	38 (16-66) vs 39 (15-69) vs 44 (20-74)	45.46 (37.11) vs 47.22 (38) vs 51.8 (39.1)	ns[k]

GCLM c.588C>T	C allele vs TT[D]	1058 vs 33	40 (16-69) vs 37 (16-60)	46.79 (38.96) vs 46.79 (38.96)	ns
	T allele vs CC[R]	328 vs 763	40 (16-66.5) vs 40 (16-71.5)	47.03 (37.3) vs 48.07 (38.39)	ns
	CC vs CT vs TT[A]	763 vs 295 vs 33	40 (16-71.5) vs 40 (16-68) vs 37 (16-60)	48.07 (38.39) vs 47.06 (37.18) vs 46.79 (38.96)	ns[k]
eNOS c.894G>T	G allele vs TT[D]	1008 vs 84	40 (16-69.25) vs 41.5 (15.25-66.5)	47.84 (38.21) vs 46.82 (37.05)	ns
	T allele vs GG[R]	520 vs 572	42 (16-70.5) vs 38 (16-68.25)	48.13 (37.29) vs 47.43 (38.86)	ns
	GG vs GT vs TT[A]	572 vs 436 vs 84	38 (16-68.25) vs 42 (16-72) vs 41.5 (15.25-66.5)	47.43 (38.86) vs 48.38 (37.37) vs 46.82 (37.05)	ns[k]
eNOS c.-786C>T	T allele vs CC[D]	904 vs 151	39 (15-71.25) vs 42 (20-62)	47.99 (39) vs 45.86 (32.55)	ns
	C allele vs TT[R]	618 vs 437	40 (18-69) vs 38 (13-69)	47.66 (36.05) vs 47.72 (40.95)	ns
	CC vs CT vs TT[A]	151 vs 467 vs 437	42 (20-62) vs 40 (17-73.5) vs 38 (13-69)	45.86 (32.55) vs 48.24 (37.13) vs 47.72 (40.95)	ns[k]
CYBA c.214T>C	C allele vs TT[D]	944 vs 133	40 (16-68) vs 46 (16-80)	47.47 (38.33) vs 50.56 (37.5)	ns
	T allele vs CC[R]	613 vs 464	40 (14-71) vs 40 (16.75-68)	47.14 (37.18) vs 48.8 (39.58)	ns

	CC vs CT vs TT[A]	464 vs 480 vs 133	40 (16.75-68) vs 40 (13.75-68) vs 46 (16-80)	48.8 (39.58) vs 46.2 (37.07) vs 50.56 (37.5)	ns[k]
CYBA c.-932G>A	G allele vs AA[D]	893 vs 177	40 (16-71) vs 41 (17-172)	48.39 (35.13) vs 46.36 (35.13)	ns
	A allele vs GG[R]	687 vs 383	39 (16-67.5) vs 44 (19.5-74)	46.27 (37.23) vs 51.25 (39.47)	ns
	GG vs AG vs AA[A]	383 vs 510 vs 177	44 (19.5-74) vs 38 (14.25-68) vs 41 (17-172)	51.25 (39.47) vs 46.24 (37.96) vs 46.36 (35.13)	ns[k]
GRS	1~2 risk alleles vs 7~8 risk alleles	66 vs 213	31.5 (13.5-53.75) vs 44 (22-68)	38.24 (31.11) vs 49.81 (34.8)	**0.0181**[d]
	1~2 risk alleles vs 9~12 risk alleles	66 vs 55	31.5 (13.5-53.75) vs 52 (19-86)	38.24 (31.11) vs 56.85 (42.1)	**0.0174**[d]

ns: non significant; GRS: genetic risk score; [k] - Kruskal-Wallis rank sum test; [d] - Dunn's test; [B]-significant after Bonferroni correction; [D]-dominant model, [R]-recessive model, [A]-additive model; T allele = TT genotype + CT genotype; C allele = CC genotype + CT genotype; A allele = AA genotype + AG genotype; G allele = GG genotype + AG genotype.

Table S2. Genotyping conditions. Primers and probes sequences**.**

SNP	Probes, primers, other reagents		Thermal profile
**5′ Nuclease Real-Time PCR assay**			
rs1799983<br>NOS3 c.894T>G	T: FAM- CCAGATGATCCCCCAGAACTCTTCC -BHQ1	50 nM	95 °C for 10 min
	G: HEX- CAGATGAGCCCCCAGAACTCTTCCT - BHQ1	50 nM	95 °C for 15 s
	F: 5′-CATTCAGCACGGCTGGA –3′	0.5μM	64 °C for 15 s
	R: 5′- ACCTCAAGGACCAGCTC -3′	0.5μM	repeat 60 times
rs2070744<br>NOS3 c.-786T>C	C: FAM- TCCCTGGCCGGCTGACCCTGCCTCA -BHQ1	50 nM	95 °C for 10 min
	T: HEX- CTTCCCTGGCTGGCTGACCCTGCCTC - BHQ1	50 nM	95 °C for 20 s
	F: 5′- TCCCACCAGGGCATCAA –3′	0.5μM	68 °C for 20 s
	R: 5′- CCCTGTCATTCAGTGACG -3′	0.5μM	repeat 60 times
rs41303970<br>GCLM c.-590C>T	C: FAM- CTGAACGCCGGGAGACCTCACCA -BHQ1	50 nM	95 °C for 10 min
	T: HEX- CCTGAACGCTGGGAGACCTCACCA - BHQ1	50 nM	95 °C for 30 s
	F: 5′- CAGGCTGCCCTTTAAAGAG –3′	0.5μM	64 °C for 30 s
	R: 5′- CCGCCTGGTGAGGTAGA -3′	0.5μM	repeat 60 times
rs2333227<br>MPO c.-463G>A	G: FAM- CTGAGGCGGGTGGATCACTTGA -BHQ1	50 nM	95 °C for 10 min
	A: HEX- CTGAGGCAGGTGGATCACTTGAG - BHQ1	50 nM	95 °C for 30 s
	F: 5′- CACACAATGGTGAGCTGAGA –3′	0.5μM	64 °C for 30 s
	R: 5′- TTTGTATTTTTCCTTAGGCAAGAAGC -3′	0.5μM	repeat 60 times
rs4880<br>SOD2 c.47T>C	T: FAM- CTGGCTCCGGTTTTGGGGTATCT -BHQ1	50 nM	95 °C for 10 min
	C: HEX- TGGCTCCGGCTTTGGGGTATC - BHQ1	50 nM	95 °C for 30 s
	F: 5′- CTTTCTCGTCTTCAGCACCA –3′	0.5μM	62 °C for 30 s
	R: 5′- CGCGTTGATGTGAGGTTC -3′	0.5μM	repeat 60 times

rs662<br>PON1 c.575A>G	A: FAM- CTACTTACAATCCTGGGAGATGT -BHQ1	50 nM	95 °C for 10 min
	G: HEX- TACTTACGATCCTGGGAGATGTA - BHQ1	50 nM	95 °C for 30 s
	F: 5′- GTTGCTGTGGGACCTGA –3′	0.5µM	55 °C for 30 s
	R: 5′- AGACAACATACGACCACG -3′	0.5µM	repeat 60 times
rs9932581<br>CYBA c.-932A>G	A: FAM-GGCAGTAATGCTGGT-BHQ1	50 nM	95 °C for 10 min
	G: HEX-GGCAGCAATGCTGGT-BHQ1	50 nM	95 °C for 30 s
	F: 5′-CTGGAATGGTGGCAGGAGT-3′	0.5µM	56 °C for 1 min
	R: 5′- CGGGATGCTGGTTTACGAA-3′	0.5µM	repeat 60 times
**PCR/RFLP assay**			
rs4673<br>CYBA c.214C>T	F: 5′-TGCTTGTGGGTAAACCAAGG-3′	0.5µM	
	R: 5′-CACTTACCTCAGTGTTTTTCC-3′	0.5µM	95°C for 5 min
	dNTPs	800 µM	95 °C for 30 s
	10x conc. PCR buffer (Thermo Scientific)	2.0 µl	55 °C for 30 s
	$MgCl_2$	1.5 µM	72 °C for 30 s
	Taq DNA polymerase (Thermo Scientific)	0.25 U	repeat 60 times
	Genomic DNA	2.0 µl	72 °C for 10 min
	Total volume	20 µl	
	PCR product of 353 bp digested with a restriction enzyme RsaI (Fermentas) which resulted in fragments: T allele: 160 bp and 193 bp and C allele: non-digested		37°C for 1 h
